# Metallothionein 3 Is a Hypoxia-Upregulated Oncogene Enhancing Cell Invasion and Tumorigenesis in Human Bladder Carcinoma Cells

**DOI:** 10.3390/ijms20040980

**Published:** 2019-02-23

**Authors:** Ke-Hung Tsui, Chen-Pang Hou, Kang-Shuo Chang, Yu-Hsiang Lin, Tsui-Hsia Feng, Chiu-Chun Chen, Yi-Syuan Shin, Horng-Heng Juang

**Affiliations:** 1 Department of Urology, Chang Gung Memorial Hospital-Linkou, Kwei-Shan, Tao-Yuan 33302, Taiwan; t2130@cgmh.org.tw (K.-H.T.); glucose1979@gmail.com (C.-P.H.); linyh@doctorvoice.org (Y.-H.L.); 2 Graduate Institute of Clinical Medical Science, College of Medicine, Chang Gung University, Kwei-Shan, Tao-Yuan 33302, Taiwan; 3 Department of Anatomy, College of Medicine, Chang Gung University, Kwei-Shan, Tao-Yuan 33302, Taiwan; D0501301@stmail.cgu.edu.tw; 4 Graduate Institute of Biomedical Sciences, College of Medicine, Chang Gung University, Kwei-Shan, Tao-Yuan 33302, Taiwan; 5 School of Nursing, College of Medicine, Chang Gung University, Kwei-Shan, Tao-Yuan 33302, Taiwan; thf@mail.cgu.edu.tw; 6 Department of Medicine, College of Medicine, Chang Gung University, Kwei-Shan, Tao-Yuan 33302, Taiwan; a5880018@gmail.com (C.-C.C.); a0956910758@gmail.com (Y.-S.S.)

**Keywords:** metallothionein 3, bladder, tumorigenesis, NDRG1, MASPIN, hypoxia

## Abstract

Metallothioneins have been viewed as modulators in a number of biological regulations regarding cancerous development; however, the function of metallothionein 3 (*MT3*) in bladder cancer is unexplored. We determined the regulatory mechanisms and potential function of MT3 in bladder carcinoma cells. Real-Time Reverse Transcriptase-Polymerase Chain Reaction (RT-qPCR) assays revealed that TSGH-8301 cells expressed more *MT3* levels than RT-4, HT1376, and T24 cells. Immunoblot and RT-qPCR assays showed that arsenic (AS_2_O_3_) treatments enhanced the gene expression of *MT3*. Hypoxia induced *HIF-1α*, *HIF-2α*, and *MT3* expression; furthermore, HIF-2α-knockdown attenuated hypoxic activation on *MT3* expression. Ectopic overexpression of *MT3* increased cell proliferation, invasion, and tumorigenesis significantly in T24 and HT1376 cells in vitro and in vivo; however, *MT3*-knockdown in TSGH-8301 cells had the reverse effect. Moreover, knockdown of *MT3* enhanced arsenic-induced apoptosis determined by the Annexin V-FITC apoptosis assay. *MT3*-overexpression downregulated the gene expressions of N-myc downstream regulated gene 1 (*NDRG1*), N-myc downstream regulated gene 2 (*NDRG2*), and the mammary serine protease inhibitor (*MASPIN*) in HT1376 and T24 cells, whereas *MT3*-knockdown in TSGH-8301 cells had the opposite effect. The experiments indicated that *MT3* is an arsenic- and hypoxia-upregulated oncogene that promotes cell growth and invasion of bladder carcinoma cells via downregulation of *NDRG1*, *NDRG2*, and *MASPIN* expressions.

## 1. Introduction

Bladder cancer has become the ninth most common worldwide cancer and the sixth most seen malignancy in the United States, according to an epidemiologic statistics report published in 2017 [1,2]. Although many tumor markers associated with the development of bladder cancer have been well recognized, the rates of mortality and recurrence of bladder cancer are still high [3,4].

Metallothioneins (MTs) are a class of low-molecular weight metal-binding proteins, which were first identified from the cortex of a horse kidney in 1957. MTs were regarded as modulators, regulating several biological processes including cellular proliferation, differentiation, invasion, and carcinogenesis [5,6,7], with four main isoforms: Metallothionein 1 (*MT1*) (subtypes A, B, E, F, G, H, L, M, X), metallothionein 2 (*MT2*), metallothionein 3 (*MT3*), and metallothionein 4 (*MT4*). The cluster of *MT* genes are located on the human chromosome 16q12-22 with a genome expansion of about 66 Kb [8]. Several in vivo studies found that trace elements and hypoxia modulated the expressions of MTs in mammalian cells [9,10,11]. Unlike *MT1* and *MT2*, the *MT3* isoform has been a subject of limited understanding.

Initially, *MT3*, known as a growth inhibitory factor, was identified as having a very limited distribution in normal tissues, and was regarded as a brain-specific MT family member with a neuronal protective function. However, later studies found that *MT3* was also expressed in other peripheral organs of mammals [12,13]. Although the mechanisms of *MT3* in cancer tumorigenesis have not been established clearly, previous studies have suggested that potentially, *MT3* can be a tumor marker for early detection of prostate and bladder cancer [14,15,16]. Interestingly, the analysis of a comparative toxicogenomics database indicated that MT3 is regarded as the cancer-associated arsenic-interacting gene in the bladder [17]. Meanwhile, *MT3* gene expression was upregulated in arsenic-transformed human urothelial cells and arsenic-treated prostate carcinoma cells [15,18].

N-myc downstream regulated genes (NDRGs), a family of proteins consisting of four members (N-myc downstream regulated gene 1 (*NDRG1*), N-myc downstream regulated gene 2 (*NDRG2*), N-myc downstream regulated gene 3 (*NDRG3*), and N-myc downstream regulated gene 4 (*NDRG4*)), play important functional roles in cancer biology, namely in tumor suppression, metastatic suppression, and oncogenesis [19]. *NDRG1*, which is normally present in human epithelial cells, has regulatory biologic effects on many cancer cells, including bladder and prostate [20]. Previous studies have illustrated *NDRG1* as a downstream gene of *MT3* in prostate carcinoma cells [15]. However, the effects of *MT3* on the expressions of NDRG family genes in bladder carcinoma cells have not been evaluated yet.

In this study, we determined the expressions of *MT3* in bladder carcinoma cells and bladder tissues, and examined the regulatory mechanisms and potential function of *MT3* in bladder carcinoma cells.

## 2. Results

### 2.1. Arsenic and Hypoxia Upregulate Metallothionein 3 (MT3) Expression in Bladder Carcinoma Cells

**Figure 1:**
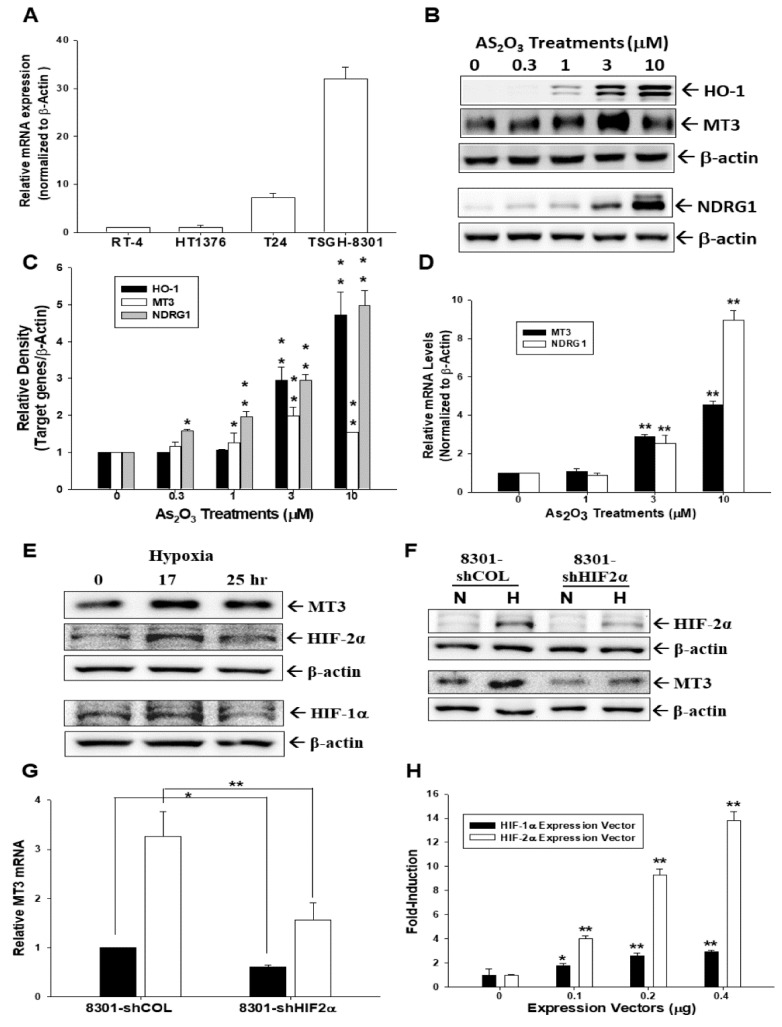

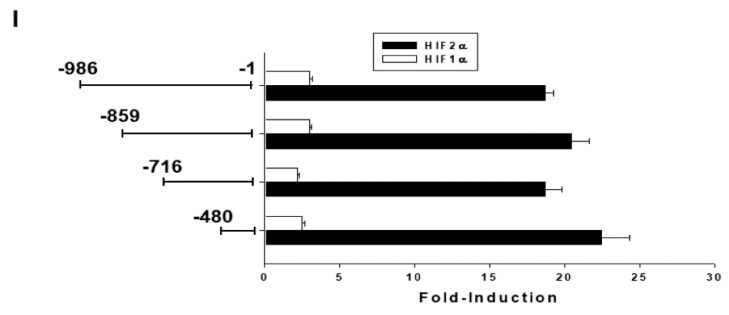
Gene expression of metallothionein 3 (*MT3*) in human bladder carcinoma cells is upregulated by arsenic and hypoxia. (**A**) Total RNA was extracted from each different bladder cell line for RT-qPCR; (**B**) T24 cells were treated with various concentrations of As_2_O_3_ for 24 h. Cells were lysed and HO-1, MT3, N-myc downstream regulated gene 1 (NDRG1), and β-actin were determined by immunoblotting; (**C**) quantitative analysis was done by determining the intensity of each band of the target gene and β-actin from three independent experiments. Data are presented as the fold-induction of the relative density of the target gene/β-actin (±SE, *n* = 3) in relation to the control solvent-treated group (* *p* < 0.05, ** *p* < 0.01); (**D**) T24 cells were treated with various concentrations of As_2_O_3_ for 24 h. Total RNA was extracted for RT-qPCR (** *p* < 0.01); (**E**) TSGH-8301 cells were cultured at a hypoxic condition in different periods. Cells were lysed, and MT3, HIF-1α, HIF-2α, and β-actin were determined by immunoblotting; (**F**) HIF-2α-knockdown TSGH-8301 (8301-shHIF2α) and mock-knockdown (8301-shCOL) cells were cultured at hypoxic or normoxic conditions for 24 h. Cells were lysed and MT3, HIF-2α, and β-actin were determined by immunoblotting; (**G**) HIF-2α-knockdown TSGH-8301 (8301-shHIF-2α) and mock-knockdown (8301-shCOL) cells were cultured at normoxic (black bars) or hypoxic (white bars) conditions for 16 h. Total RNA was extracted for RT-qPCR. Data are presented as the fold-induction of the mRNA levels of MT3/β-actin (±SE, *n* = 3) in relation to the mRNA levels of 8301-shCOL cells cultured at normoxic conditions (* *p* < 0.05, ** *p* < 0.01); (**H**) TSGH-8301 cells were cotransfected with an MT3 reporter vector and various concentrations of HIF-1α (black bars) or HIF-2α (white bars) expression vectors as indicated. Data are presented as the mean percentage ±SE (*n* = 6) of luciferase activity in relation to the control group (* *p* < 0.05, ** *p* < 0.01); (**I**) relative luciferase activity of reporter vectors containing different fragments from the MT3 promoter, as shown. The MT3 reporter vector-transfected HT1376 cells were cotransfected with the HIF-1α (white bars) or HIF-2α (black bars) expression vectors for 72 h. Luciferase activity was fold-induced (±SE, *n* = 6) in relation to the cotransfected pcDNA3 expression vector group.

The *MT3* mRNA levels in several lines of cultured bladder cells (RT4, HT1376, T24, and TSGH-8301) were compared. Results of RT-qPCR assays revealed that TSGH-8301 cells had the highest levels of *MT3* among the four bladder carcinoma cell lines (Figure 1A). Results of immunoblot assays showed that arsenic upregulated *HO-1*, *MT3*, and *NDRG1* protein levels in T24 cells (Figure 1B). Results of quantitative analyses from three independent experiments are present in Figure 1C. Results of RT-qPCR revealed that arsenic treatment-induced *MT3* and *NDRG1* gene expressions were dosage-dependent (Figure 1D). Further immunoblot assays indicated that 17 h of hypoxia upregulated *HIF-1α*, *HIF-2α*, and *MT3* protein levels in TSGH-8301 cells (Figure 1E); moreover, HIF-2α-knockdown in TSGH-8301 cells blocked *HIF-2α* and *MT3* expressions under the hypoxic condition determined by immunoblotting (Figure 1F) and RT-qPCR (Figure 1G) assays. Results of reporter assays showed that transient overexpression of *HIF-1α* and *HIF-2α* induced promoter activity of the human *MT3* gene (Figure 1H); in addition, 5′-delation report assays showed that *HIF-1α* and *HIF-2α* induced *MT3* promoter activity was dependent on the 5′-flanking DNA fragment (−1 to −480) (Figure 1I).

### 2.2. Effects of Ectopic Overexpression of MT3 on Proliferation and Invasion of Bladder Carcinoma HT1376 Cells

**Figure 2:**
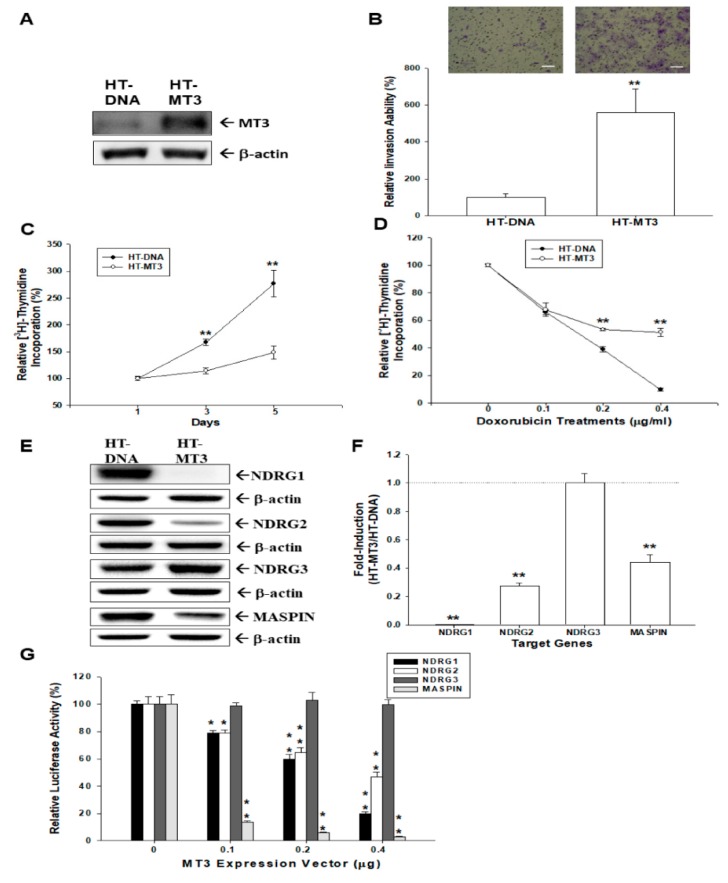
Effects of MT3 on proliferation, invasion, and expressions of *NDRG1*, *NDRG2*, *NDRG3*, and *MASPIN* (mammary serine protease inhibitor) genes in HT1376 cells. (**A**) Ectopic overexpression of *MT3* was determined by an immunoblot assay; (**B**) the invasive ability of cells was determined by in vitro Matrigel invasion assays. Data are presented as the mean percentage (±SE, *n* = 3) of invasion ability in relation to that of the HT−DNA cell group (** *p* < 0.01). The scale bar is 50 μm; (**C**) proliferation rates in HT−MT3 (black circle) and HT−DNA (white circle) cells were determined by ^3^H-thymidine incorporation assays (** *p* < 0.01); (**D**) *MT3* modulates the effect of doxorubicin on the proliferation of HT1376 cells. HT−DNA (●) and HT−MT3 (○) cells were treated with varying concentrations of doxorubicin as indicated for 48 h. Each point on the curve represents the mean percentage (±SE, *n* = 6) relative to day 1 (** *p* < 0.01) Ectopic overexpression of *MT3* affects the expressions of *NDRG1*, *NDRG2*, *NDRG3*, and *MASPIN* genes in HT1376 cells determined by immunoblot (**E**) and RT-qPCR (**F**) assays (** *p* < 0.01); (**G**) *NDRG1*, *NDRG2*, *NDRG3*, and *MASPIN* reporter vectors were individually cotransfected with different concentrations of the *MT3* expression vector into HT1376 cells for 72 h. Data are expressed as the mean percentage ±SE of luciferase activity relative to the mock-transfected group (* *p* < 0.05, ** *p* < 0.01).

A human *MT3* expression vector was transfected into bladder carcinoma HT1376 cells to investigate the role of *MT3* in proliferation and invasion. Results of the immunoblot assay confirmed the ectopic overexpression of *MT3* in HT1376 (HT−MT3) cells (Figure 2A). Matrigel invasion assays revealed that HT−MT3 cells expressed markedly a higher invasive capacity than HT−DNA cells (Figure 2B). [^3^H]thymidine incorporation assays revealed that the numbers of HT−MT3 cells increased 2.82 folds after five days of incubation. However, HT−DNA cells increased only by 1.45-folds (Figure 2C). Furthermore, [^3^H]thymidine incorporation assays revealed that MT3-overexpressed HT1376 cells attenuated the effect of doxorubicin on cell proliferation. Doxorubicin (0.4 μg/mL) blocked 93% of proliferation of HT−DNA cells, whereas proliferation of HT−MT3 cells was decreased only by 49% after 48 h of treatment (Figure 2D).

### 2.3. Ectopic Overexpression MT3 Modulates N-myc Downstream Regulated Gene 1 (NDRG1), NDRG2, NDRG3, and MASPIN Gene Expressions in Bladder Carcinoma HT1376 Cells

Further studies of immunoblot (Figure 2E), RT-qPCR (Figure 2F), and reporter (Figure 2G) assays showed that *MT3*-overexpression (HT-MT3) cells present markedly lower levels of *NDRG1*, *NDRG2*, and *MASPIN* gene expressions than mock-overexpression (HT-DNA) cells. HT-MT3 and HT-DNA cells did not display significant differences in the expressions of the *NDRG3* gene.

### 2.4. Effect of MT3-Knockdown on Proliferation and Invasion of Bladder Carcinoma TSGH-8301 Cells

**Figure 3:**
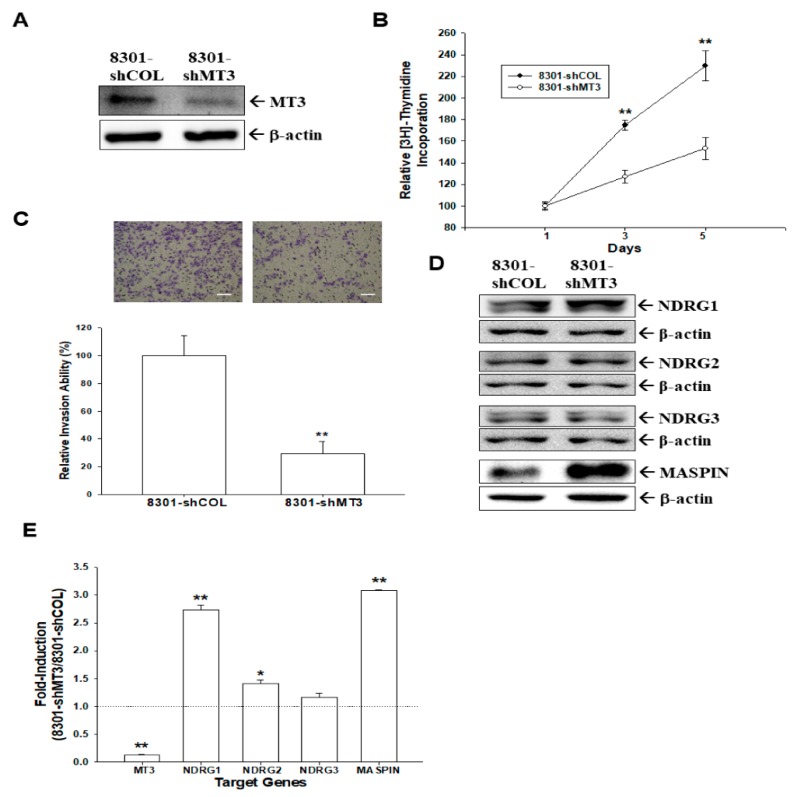
*MT3*-knockdown enhances proliferation and invasion in TSGH-8301 cells. (**A**) Expressions of *MT3* in mock-knockdown TSGH-8301 (8301-shCOL) and *MT3*-knockdown TSGH-8301 (8301-shMT3) cells were determined by an immunoblot assay; (**B**) proliferation rates in 8301-shCOL (●) and 8301-shMT3 (○) cells were determined by ^3^H-thymidine incorporation assays (** *p* < 0.01). Each point on the curve represents the mean percentage (±SE, *n* = 6) relative to day 1; (**C**) invasion ability of cells was determined by in vitro Matrigel invasion assays (** *p* < 0.01). Data are presented as the mean percentage (±SE) of the invasion ability in relation to that of the 8301-shCOL cell group. The scale bar is 50 μm. Expressions of *NDRG1*, *NDRG2*, *NDRG3*, and *MASPIN* in 8301-shCOL and 8301-shMT3 cells were determined by immunoblot (**D**) and RT-qPCR (**E**) assays (* *p* < 0.05, ** *p* < 0.01).

Using immunoblot assays, we confirmed that the expression of *MT3* was only 10% in *MT3*-knockdown TSGH-8301 (8301-shMT3) cells compared to mock-knockdown (8301-shCOL) cells (Figure 3A). [^3^H]thymidine incorporation assays revealed a 2.30-fold increase in the number of 8301-shCOL cells after five days of incubation. However, the number of 8301-shMT3 cells increased only by 1.54 folds (Figure 3B). Matrigel assays indicated that knockdown of *MT3* resulted in a 64% decrease in invasion capacity compared with 8301-shCOL cells (Figure 3C). These results suggested that knockdown of *MT3* in bladder carcinoma TSGH-8301 cells, which have higher endogenous *MT3* levels, blocked cell proliferation and invasion. Further studies of immunoblot (Figure 3D) and RT-qPCR (Figure 3E) assays showed that *MT3*-knockdown (8301-shMT3) cells exhibited markedly higher levels of *NDRG1* and *MASPIN* gene expressions than mock-knockdown (8301-shCOL) cells. The expresion of *NDRG2* was induced a little but significantly after knockdown of *MT3*. However, *MT3*-knockdown did not significantly affect *NDRG3* gene expression in TSGH-8301 cells.

### 2.5. Effect of Overexpression of MT3 on Proliferation and Invasion of Bladder Carcinoma T24 Cells

The ectopic expression of *MT3* in T24 cells was confirmed using immunoblot assays (Figure S1A) and Matrigel invasion assays indicated that the invasive ability of T24−MT3 cells was about 3.78-fold higher than that of T24−DNA cells (Figure S1B). [^3^H]thymidine incorporation assays revealed a 5.06-fold increase in the number of T24−MT3 cells after five days of incubation. However, the number of T24−DNA cells increased only by 3.32 folds (Figure S1C).

### 2.6. Overexpression of MT3 Downregulates NDRG1, NDRG2, and MASPIN Gene Expressions in Bladder Carcinoma T24 Cells

The results of immunoblot (Figure S1D), RT-qPCR (Figure S1E), and reporter (Figure S1F) assays showed that *MT3*-overexpression (T24−MT3) cells had markedly lower levels of *NDRG1*, *NDRG2*, and *MASPIN* gene expressions than mock-overexpression (T24−DNA) cells.

### 2.7. Effects of Ectopic Overexpression of MT3 on Tumorigenesis of Bladder Carcinoma T24 cells

**Figure 4:**
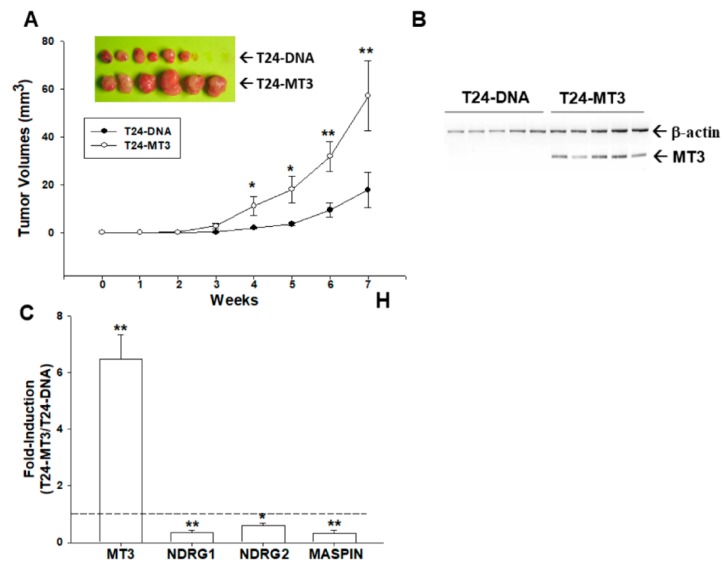
Effects of *MT3* on tumorigenesis in T24 cells. (**A**) Nude mice were inoculated subcutaneously with T24−DNA (●) or T24−MT3 (○) cells. Tumor size (mm^3^) was measured with a Vernier caliper at the indicated days (* *p* < 0.05, ** *p* < 0.01); (**B**) whole-cell lysates of randomly selected tumor samples from T24−DNA and T24−MT3 groups were subjected to immunoblot-blot analysis; (**C**) quantitative analysis of RT-qPCR is presented as the mean of the fold-induction of mRNA levels of *MT3*, *NDRG1*, *NDRG2*, and *MASPIN* (±SE, *n* = 3) relative to the mock-transfected xenograft groups (* *p* < 0.05, ** *p* < 0.01).

The effect of *MT3* on tumor growth in vivo was evaluated using xenografts in BALB/cAnN-Foxn1^NU^ mice. Tumors generated from T24−DNA cells grew slower than those derived from T24−MT3 cells. Additionally, tumors generated from T24−DNA cells were approximately 32% smaller than tumors generated from T24−MT3 cells (18.01 ± 7.38 vs. 57.30 ± 14.53 mm^3^) after 7 weeks of growth (Figure 4A). Thus, ectopic overexpression of *MT3* enhanced cell proliferation, invasion, and tumorigenesis of T24 cells. The results of immunoblot assays confirmed that *MT3* was overexpressed in the xenograft tumors, which were inoculated by T24−MT3 cells (Figure 4B). Moreover, the results of RT-qPCR assays revealed that overexpression of *MT3* blocked *NDRG1*, *NDRG2*, and *MASPIN* mRNA levels in xenograft tumors (Figure 4C).

### 2.8. MT3 Modulates Cell Apoptosis Induced by As_2_O_3_ in Bladder Carcinoma Cells

**Figure 5:**
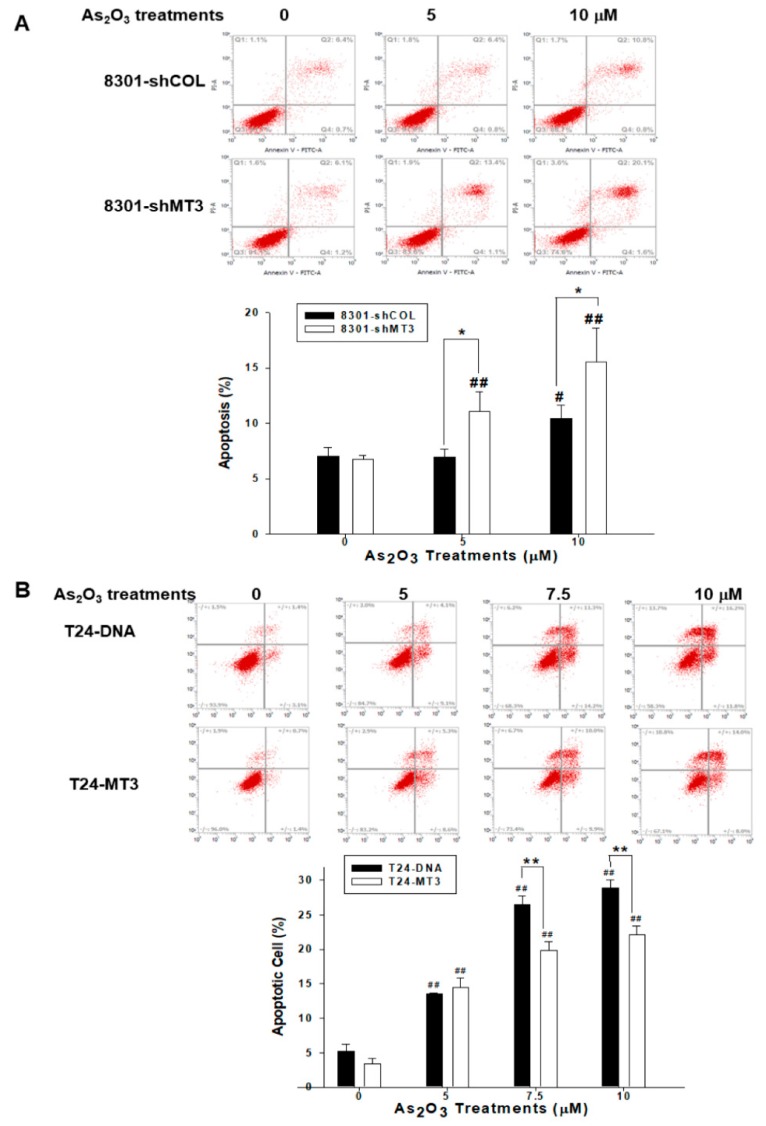
Modulation of MT3 on As_2_O_3_-induced cell apoptosis in bladder carcinoma cells. (**A**) TSGH-8301 (8301-shCOL and 8301-shMT3) and (**B**) T24 (T24−DNA and T24−MT3) cells after treatments with different concentrations of As_2_O_3_ for 24 h. The fluorescence intensity for Annexin V-FITC in conjunction with PI staining to distinguish among early apoptotic, late apoptotic, and necrotic cells was determined by using flow cytometry. Data are presented as the percentage of apoptotic cells after treatments (* and # *p* < 0.05, ** and ## *p* < 0.01).

To distinguish among early apoptotic, late apoptotic, and necrotic cells of bladder carcinoma, we used Annexin V-FITC with propidium iodide (PI) staining. The flow cytometry of fluorescence intensity for Annexin V-FITC and PI staining in 8301-shCOL and 8301-shMT3 cells after treatments with various concentrations of As_2_O_3_ for 24 h revealed that *MT3*-knockdown TSGH-8301 (8301-shMT3) cells significantly enhanced cell apoptosis induced by As_2_O_3_ in comparison to mock-knockdown TSGH-8301 (8301-shCOL) cells (Figure 5A). On the contrary, ectopic overexpression of *MT3* in T24 (T24−MT3) cells significantly attenuated cell apoptosis induced by As_2_O_3_ in comparison to mock-overexpression T24 (T24−DNA) cells (Figure 5B).

## 3. Discussion

Metallothioneins (MTs), a class of metal-binding proteins characterized by high-cysteine content and low-molecular weight, may provide protection against metal toxicity and oxidative stress [13,21]. Early reports found that an increased expression of MTs was related to cancer in the breast, colon, kidney, liver, skin, lung, nasopharynx, ovary, prostate, thyroid, and urinary bladder, whereas hepatocellular carcinoma and liver adenocarcinoma produced a lower level of MT expression [6]. Furthermore, defects in the MT function or expression may lead to a malignant transformation of cancer, including bladder cancer, because MTs play an important role in transcription factor regulation [22,23,24]. The *MT3* isoform possesses a unique sequence of eight amino acids which is not present in any other members of the MT gene family [25]. In addition, in nerve-derived cell cultures, *MT3* possesses an inhibitory activity for neural cell growth, which is not duplicated by other MT isoforms [26]. However, some studies revealed that *MT3* was found in normal prostate and renal tissues with an altered expression in organ-derived malignancies [27,28]. A prior study suggested that *MT3* might be an effective biomarker for bladder cancer although the biologic functions of *MT3* have not been fully understood [14]. Our study suggests that *MT3* expression in the bladder cell lines could be dependent on the cell type but not relevant to the extent of neoplasia in vitro. A similar result was also found in a previous study using prostate carcinoma cells [15].

An epidemiologic study indicated that arsenic pollution in water is associated significantly with the incidence of bladder cancer in Taiwan [29]. Animal studies found that *MT3* was overexpressed in tumor heterotransplants derived from arsenic-transformed human urothelial cells [30]. Ours has been the first study to reveal that arsenic can induce *HO-1*, *NDRG1* and *MT3* gene expressions in bladder carcinoma T24 cells. The results of upregulation of *MT3* by arsenic are consistent with those of a previous study on prostate carcinoma LNCaP cells [15]. Although early studies indicated that arsenic-induced metal-responsive transcription factor-1 (*MTF-1*) binds to the metal-response element (MRE) of the promoter *MT3* and *NDRG1* genes [18,31], the molecular mechanisms of arsenic on gene expressions of *MT3* and *NDRG1* in bladder carcinoma cells are still undefined. Expression of *HO-1*, a gene known to be induced in response to sodium arsenite, was used as positive control [32]. The *HO-1* expression increased as the dosage of As_2_O_3_ increased, demonstrating the effectiveness of treatment. 

Studies in mouse MEF cells and primary culture chondrocytes indicated that hypoxia induced both *MT1* and *MT2* expressions through cooperative interactions between transcription factors and *HIF-1α* or *HIF-2α*, respectively [33,34]. Although studies suggested that hypoxia upregulated *MT3* in human prostate carcinoma PC-3 cells and adipocytes, no mechanisms were illustrated in their reports [11,35]. As shown in Figure 1, the present study has been the first to indicate that upregulation of *MT3* by hypoxia in human bladder carcinoma cells is dependent on *HIF-1α* and *HIF-2α*, which are well-known to be overexpressed in bladder cancer in vivo [36].

Our findings indicated that *MT3* affects cell proliferation and invasion in bladder carcinoma cells. Results of ^3^H-thymidine incorporation assays, Matrigel invasion assays, and xenografts in mice showed that mock-transfected bladder carcinoma cells grew slower in vitro and in vivo than the *MT3*-overexpressed bladder carcinoma cells, whereas *MT3*-knockdown attenuated cell invasion of TSGH-8301 cells. These results are similar to our earlier study using prostate carcinoma cells, which showed that mock-transfected PC-3 cells grew slower in vitro and in vivo than PC-3 cells overexpressing *MT3* [15]. To the best of our knowledge, our study has been the first to provide laboratory evidence that *MT3* plays a tumor inductive role in human bladder carcinoma cells. Our study also indicated that *MT3* overexpression increased resistance to doxorubicin in HT1376 cells (Figure 2). This finding of *MT3* overexpression increasing chemotherapeutic drug resistance is in agreement with a previous study in prostate carcinoma cells [15,37]. It is possible that overexpression of *MT3* is one of the mechanisms of bladder tumor cell resistance to cancer treatment.

Studies suggested that metallothioneins could be involved in protection against toxicity, and regulate the interactive effects of metals and metalloids including arsenic [11]. This study showed that As_2_O_3_ induced more apoptosis in *MT3*-knockdown TSGH-8301 cells than mock-transfected TSGH-8301 cells. On the contrary, ectopic overexpression of *MT3* in T24 cells significantly attenuated cell apoptosis induced by As_2_O_3_ in comparison to mock-overexpression of T24 cells (Figure 5). Our results demonstrated that *MT3* might be involved in the protection against arsenic toxicity in bladder carcinoma cells. Results also suggested that *MT3* was similar to other metallothioneins involved in the intracellular defense mechanism against arsenic cytotoxicity [38].

In the present study, we found that ectopic *MT3* overexpression in HT1376 and T24 cells blocked gene expressions of *NDRG1*, *NDRG2*, and *MASPIN*, but not *NDRG3*. In vitro and in vivo studies have shown that *NDRG1*-induced expression downregulated the growth of bladder carcinoma cells [39]. Overexpression of *NDRG2* in bladder carcinoma cells inhibited cell proliferation, invasion, and tumorigenesis in vitro and in vivo; moreover, the expression of *NDRG2* correlated negatively with the tumor grade and pathologic stage of bladder cancer [40]. Mammary serine protease inhibitor (*MASPIN*), a member of the serine protease inhibitor family, inhibited cell proliferation, migration, and invasion of bladder carcinoma cells [41]. Our results suggest that decreased expressions of *NDRG1*, *NDRG2*, and *MASPIN* genes may account for the increased cell proliferation and invasiveness in bladder carcinoma cells with *MT3* stably overexpressed. A prior study identified 43 *MT3*-target genes after ectopic overexpression of *MT3* in HL-60 cells [42] although signal pathways of *MT3* on its downstream genes still need to be explored further.

## 4. Materials and Methods

### 4.1. Cell Cultures and Chemicals

The bladder transitional carcinoma cell lines, RT-4, HT1376, TSGH-8301, and T24 cells, were purchased from the Bioresource Collection and Research Center (BCRC, Hsinchu, Taiwan) as described previously [43]. The RT-4 cell line was obtained from the explant of a recurrent papillary bladder tumor [44]. The HT1376 cell line, generated from a Caucasian woman with grade 3 transitional cell bladder cancer, contained the well-differentiated human bladder carcinoma cells with high tumorigenic capability [45]. The TSGH-8301 cell line was taken from a Taiwanese well-differentiated transitional cell carcinoma [46]. The T24 cell line was poorly differentiated transitional carcinoma cells with low tumorigenic capability [47]. DAPI (4,6-diamino-2-phenylindole), bovine serum albumin (BSA), humic acid (HA), and As_2_O_3_ were obtained from Sigma-Aldrich Co. (St. Louis, MO, USA). The As_2_O_3_ stock solution was dissolved in 1 mM humic acid solution at a concentration of 100 mM, as modified from a previous study [48]. We purchased fetal calf serum (FCS) from HyClone (Logan, UT, USA), RPMI 1640 media from Invitrogen (Carlsbad, CA, USA), and Matrigel from BD Biosciences (Bedford, MA, USA).

### 4.2. Knock-down MT3 and HIF-2α

TSGH-8301 cells were transduced with control shRNA lentiviral particles-A (Sc-108080), *MT3* shRNA lentiviral particles (sc-93438-V), or *HIF-2α* shRNA lentiviral particles (sc-35316-V). Two days after transduction, the cells (8301-shCOL, 8301-shMT3, and 8301-shHIF-2α) were selected by incubation with 4 μg/mL puromycin dihydrochloride for at least another 5 generations.

### 4.3. Expression Vectors and Stable Transfection

The expression vectors of *MT3* and *HIF-1α* were cloned as described previously [15,49]. The *HIF*-2α expression vector (HA-HIF-2α-P405A/P531A-pcDNA3) was purchased from Addgene (Cambridge, MA, USA). Electroporation was conducted using an ECM 830 Square Wave Electroporation System (BTX, San Diego, CA, USA), set at 180 V (for T24 cells) or 190 V (for HT1376 cells), 70-msec pulse length, and one pulse setting. Transfected cells (T24−MT3 and HT1376−MT3) were selected by 100 μg/mL of Zeocin (Invitrogen). For construction of the mock-transfected cells (T24−DNA and HT1376−DNA), cells were transfected with a controlled pcDNA3.1/Zeo expression vector (Invitrogen) and were clonally selected in the same manner as the one described above.

### 4.4. Immunoblot Assays

For nuclear and cytoplasmic extraction, cells were cultured in an RPMI-1640 medium with 10% FCS for 48 h, and then harvested with trypsin, and washed twice with PBS. Nuclear and cytoplasmic fractions were separated using the NE-PER^TM^ Nuclear and cytoplasmic extraction kit (Thermo, Rockford, NJ, USA) as described by the manufacturer. Equal amounts of whole cell, nuclear, or cytoplasmic lysis were loaded onto a 10% SDS-polyacrylamide gel and assayed by enhanced chemiluminescence as described by the manufacturer (PerkinElmer Inc, Waltham, MA, USA). Blotting membranes were probed with antiserum of MT3 (Sigma-Aldrich Co.), heme oxygenase-1 (HO-1; Stressgen, Victoria, BC, Canada), NDRG1 (Invitrogen), NDRG2, NDRG3 (Abcam, Cambridge, UK), HIF-1α, MASPIN (BD Biosciences, San Jose, CA, USA), HIF-2α (Novus, Littleton, CO, USA), or β-actin antiserum (Millipore, Billerica, MA, USA). The intensities of different bands were analyzed using the GeneTools of ChemiGenius (Syngene, Cambridge, UK).

### 4.5. Real-Time Reverse Transcriptase-Polymerase Chain Reaction (RT-qPCR)

Total RNA was isolated using Trizol reagent, and cDNA was synthesized using a Superscript III pre-amplification system (Invitorgen) as described previously [39]. Real-time polymerase chain reactions were performed using a CFX Connect Real-Time PCR system (Bio-Rad Laboratories, Foster City, CA, USA) and PCR primers for human *MT3* (Hs00359394_g1), *MASPIN* (Hs00985283_ml), *NDRG1* (Hs00608387_ml), *NDRG2* (Hs0104515_m1), *NDRG3* (Hs00259237_m1), and *β-actin* (Hs01060665_g1) were purchased from Applied Biosystems (Foster City, CA, USA). The mean cycle threshold (*C*_t_) values were calculated for internal control and target genes as described in detail previously [50].

### 4.6. [^3^H]thymidine Incorporation Assay

The [^3^H]thymidine incorporation assay was used to measure cell proliferation as described previously [49].

### 4.7. Matrigel Invasion Assay

Cells were suspended at a density of 1 × 10^5^/100 μL in a serum-free medium and seeded into a 24-well transwell chamber with an 8-μm pore membrane. The 4% (*w*/*v*) paraformaldehyde was used to fix the cells that migrated into the Matrigel-coated transmembrane; these were then stained with a 0.1% (*w*/*v*) crystal violet solution. In order to capture images, a digital camera connected to an inverted microscope (IX71, Olympus, Tokyo, Japan) was used. By using PAX-it image analysis software, we analyzed the images following standardization of light intensity as described previously [50].

### 4.8. Annexin V-FITC Apoptosis Detection

Cell pellets were harvested after cells were treated with or without arsenic (AS_2_O_3_) for 24 h. Apoptosis detection and quantification were performed after treatments with Annexin V-FITC (BioVision Inc, Milpitas, CA, USA) and propidium iodide (PI) for 1 h using the FACSCalibur E6147 Cytometer (BD Biosciences) as described previously [51].

### 4.9. Reporter Vector Constructs and Reporter Assay

Human *MT3* (−1 to −986), *MASPIN* (−5948 to −5), *NDRG1* (−4714 to +46), *NDRG2* (−4253 to −1), and *NDRG3* (−5734 to +178) reporter vectors were constructed as described in detail previously [15,44,52]. MT3 reporter vectors (−1 to −859, −1 to −716, and −1 to −480) containing 5′-deletion fragments were synthesized by PCR using a GL2 primer and *MT3* specific primers (5′-GAGCTCTTGTTGCACAGGCTGAAG-3′, 5′-GAGCTCTTTAGTAGAGATGGGGTT-3′, and 5′-GAGCTCGGCCTCAGGCTTAGATGGTAC-3′), respectively; then, the DNA fragment was digested and cloned into the luciferase reporter vector (pGL3-Basic; Promega Bioscience, San Luis Obispo, CA, USA). Cells were seeded at a density of 10^4^ cells/well in a 24-well plate and allowed to grow for 24 h. Cells were then transfected transiently with a luciferase reporter vector for another 48 h using the X-tremeGENE HP DNA Transfection Reagent (Roche Diagnostic GmbH, Mannheim, Germany) following the protocol of the manufacturer. The relative luciferase activities were then measured and reported in relative light units (RLU).

### 4.10. Tumor Xenograft Study

This animal study was approved by the Institutional Animal Care and Use Committee of the Chang Gang University (IACUC Approval No.: CGU14-075, 9 September 2014). In this study, we purchased 4-week-old male BALB/cAnN-Foxn1^NU^ mice from the National Laboratory Animal Center, Taipei, Taiwan. These mice were under anesthesia during surgical procedures and all effort was made to minimize distress and pain. Each mouse was under intraperitoneal anesthesia when the pre-prepared T24−DNA and T24−MT3 cells were mixed (1:1) with Matrigel and injected subcutaneously into one side of the back near the shoulder of each mouse (*n* = 6). The mice were kept in a barrier facility under HEPA filtration and their health was monitored weekly during the experiment. Xenograft growth was measured by Vernier calipers at intervals as indicated, and tumor volume was calculated as volume = [π/6 × largest diameter × (smallest diameter)^2^] as described previously [43].

### 4.11. Statistical Analysis

Results are expressed as the mean ± (SE) of at least three independent replications of each experiment. Statistical significance was determined by one-way ANOVA and Student’s *t* test using the SigmaStat program for Window version 2.03 (SPSS Inc, Chicago, IL, USA). The * represents *p* < 0.05 and the ** represents *p* < 0.01.

## 5. Conclusion

Our experiments provided evidence indicating that arsenic and hypoxia upregulated *MT3* expression. Ectopic overexpression of *MT3* enhanced tumorigenesis of bladder carcinoma T24 cells in vivo. The downregulation of *NDRG1*, *NDRG2*, and *MASPIN* gene expressions could account for the enhancement of proliferative and invasive functions of *MT3* in bladder carcinoma cells. It was found that *MT3* might also participate in the protection against arsenic toxicity in bladder carcinoma cells. The results suggested that *MT3* is the oncogene in bladder cancer and ectopic overexpression of *MT3* enhances tumorigenesis of human bladder carcinoma cells.

## Supplementary Materials

Supplementary materials can be found at https://www.mdpi.com/1422-0067/20/4/980/s1.
