# Supplementary figures and images for: Metallothionein 3 Is a Hypoxia-Upregulated Oncogene Enhancing Cell Invasion and Tumorigenesis in Human Bladder Carcinoma Cells
Source: Int J Mol Sci. 2019 Feb 23;20(4):980. doi: 10.3390/ijms20040980 (PMC6413184; doi:10.3390/ijms20040980)

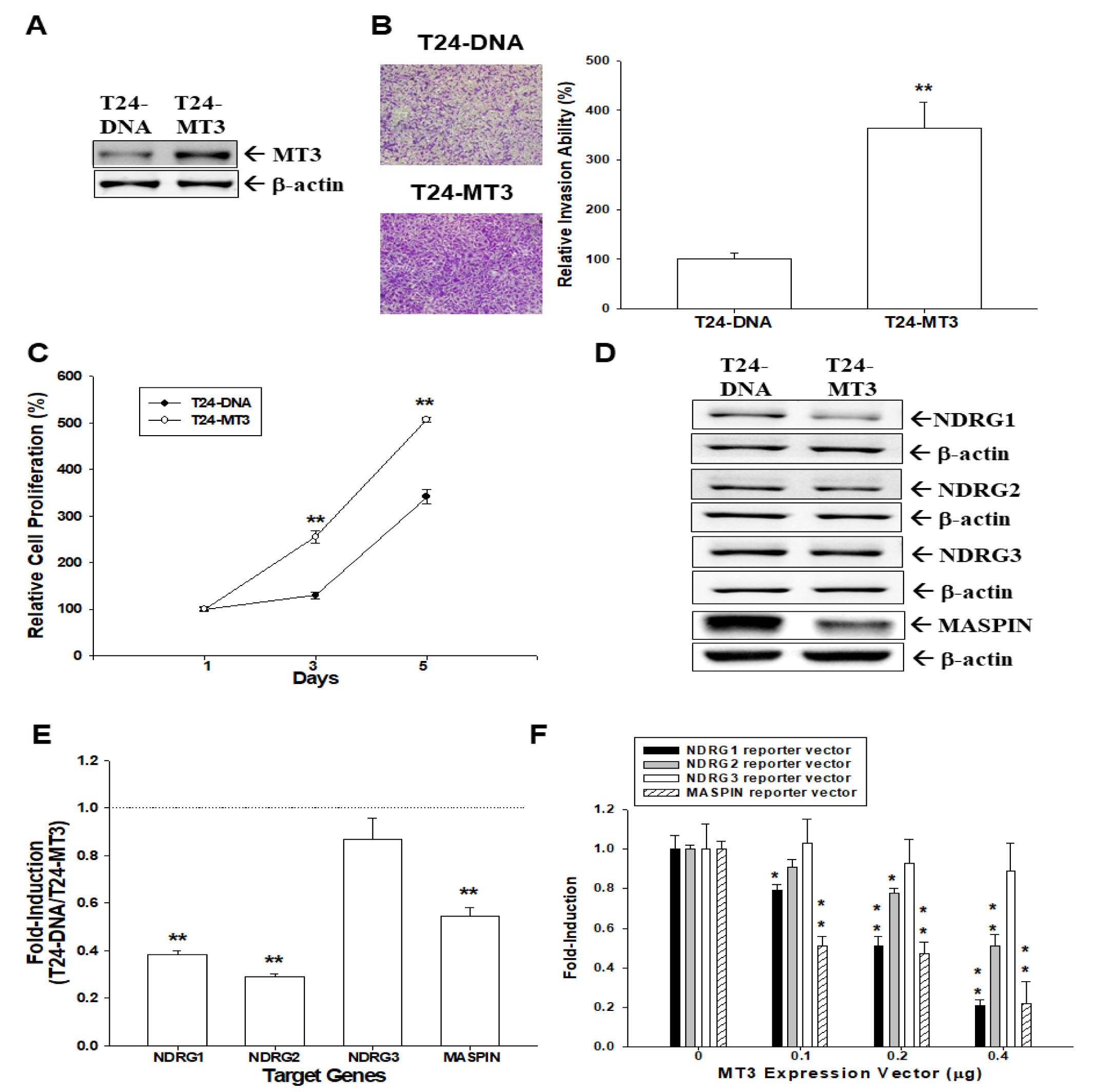

Supplement: Supplementary file 1 [file ijms-20-00980-s001.zip › ijms-444477-supplementary(1).tif]
